# Supplementary material for: Simulation models of sugary drink policies: A scoping review
Source: PLoS One. 2022 Oct 3;17(10):e0275270. doi: 10.1371/journal.pone.0275270 (PMC9529101; doi:10.1371/journal.pone.0275270)
Supplement: S1 File — (PDF) [file pone.0275270.s001.pdf]

SUPPLEMENTARY MATERIAL

1 PubMed 1
2 Embase 2
3 CINAHL 3
4 Cochrane Reviews 4
5 Communication and Mass Media Complete 6
6 PsycInfo 6
7 Scopus 7

Search Strategies

The search strategy was conducted in PubMed using keyword and MeSH combinations. Other database search strategies used textwords and controlled vocabulary where applicable.

1 PubMed

searched June 25, 2020

1704 results

updated search June 10, 2021 172 results

("Life Tables"[Mesh] OR life table OR life tables OR life-table OR life-tables OR "Computer Simulation"[Mesh] OR "Quality-Adjusted Life Years"[Mesh] OR "Models, Theoretical/analysis"[Mesh] OR "Models, Theoretical/economics"[Mesh] OR "Models, Theoretical/epidemiology"[Mesh] OR "Models, Theoretical/mortality"[Mesh] OR "Models, Theoretical/prevention and control"[Mesh] OR "Models, Theoretical/statistics and numerical data"[Mesh] OR "Systems Analysis"[Mesh] OR "Markov Chains"[Mesh] OR "Cost-Benefit Analysis"[Mesh] OR "Models, Economic"[Mesh] OR computer simulation OR computer simulations OR computer model OR computer models OR computerized model OR computerized models OR QALY OR QALYs OR quality-adjusted life year OR quality-adjusted life years OR quality adjusted life year OR quality adjusted life years OR adjusted life year OR adjusted life years OR healthy years equivalent OR healthy years equivalents OR theoretical model OR theoretical models OR mathematical model OR mathematical models OR systems analysis OR systems analyses OR systems oriented approach OR systems oriented approaches OR system dynamics analysis OR system dynamics analyses OR systems approach OR systems approaches OR systems thinking OR agent-based modeling OR agent-based model OR agent based model OR agent based modeling OR markov chain OR markov process OR markov processes OR cost-benefit analysis OR cost-benefit analyses OR cost benefit analysis OR cost benefit analyses OR cost-benefit data OR cost benefit data OR cost-utility analysis OR cost-utility analyses OR cost utility analysis OR cost utility analyses OR economic evaluation OR economic evaluations OR cost benefit OR "costs and benefits" OR "benefits and costs" OR cost-effectiveness analysis OR cost-effectiveness analyses OR economic model OR economic models OR individual based

model OR individual based modeling OR individual-based model OR individual-based modeling OR microsimulation OR microsimulation model OR microsimulation modeling OR simulation model OR simulation modeling OR cohort model OR cohort modeling OR compartmental model OR compartmental modeling OR markov model OR markov modeling) AND ("Health Policy"[Mesh] OR "Nutrition Policy"[Mesh] OR "Health Promotion"[Mesh] OR "Diet, Healthy"[Mesh] OR "Public Health/economics"[Mesh] OR "Public Health/epidemiology"[Mesh] OR "Public Health/methods"[Mesh] OR "Public Health/mortality"[Mesh] OR "Public Health/prevention and control"[Mesh] OR "Public Health/statistics and numerical data"[Mesh] OR health policies OR health policy OR nutrition policy OR nutrition policies OR food policy OR food policies OR nutrition guideline OR nutrition guidelines OR dietary guideline OR dietary guidelines OR public health[tiab] OR population health[tiab] OR health benefits[tiab] OR policy scenario OR policy impact OR policy intervention) AND ("Carbonated Beverages"[Mesh] OR "Sugar-Sweetened Beverages"[Mesh] OR ((ssb[tiab] OR ssbs[tiab]) AND (beverage*[tiab] OR drink*[tiab] OR juic*[tiab] OR soda*[tiab])) OR sweet drink OR sweet drinks OR sweet beverage OR sweet beverages OR sweetened drink OR sweetened drinks OR sweetened beverage OR sweetened beverages OR sugary drink OR sugary drinks OR sugary beverage OR sugary beverages OR carbonated beverage OR carbonated beverages OR carbonated drink OR carbonated drinks OR soft drink OR soft drinks OR soda[tiab] OR sodas[tiab] OR soda-pop OR soda pop OR soda pops OR cola[tiab] OR coca-cola OR flavored water OR flavored waters OR flavoured water OR flavoured waters OR juice OR juices OR fruit-flavored drink OR fruit-flavored drinks OR fruit-flavoured drink OR fruit-flavoured drinks OR fruit-flavored beverage OR fruit-flavored beverages OR fruit-flavoured beverage OR fruit-flavoured beverages OR drink[tiab] OR drinks[tiab] OR beverage[tiab] OR beverages[tiab]) NOT (editorial[pt] OR letter[pt] OR comment[pt])

2 Embase

1093 results

searched June 25, 2020

updated search June 10, 2021 123 results

('life table'/exp OR 'life table' OR 'life tables'/exp OR 'life tables' OR 'computer simulation'/exp OR 'computer simulation' OR 'computer simulations' OR 'computer model'/exp OR 'computer model' OR 'computer models' OR 'computerized model' OR 'computerized models' OR 'qaly'/exp OR qaly OR qalys OR 'quality-adjusted life year'/exp OR 'quality-adjusted life year' OR 'quality-adjusted life years'/exp OR 'quality-adjusted life years' OR 'quality adjusted life year'/exp OR 'quality adjusted life year' OR 'quality adjusted life years'/exp OR 'quality adjusted life years' OR 'adjusted life year' OR 'adjusted life years' OR 'healthy years equivalent' OR 'healthy years equivalents' OR 'theoretical model'/exp OR 'theoretical model' OR 'theoretical models' OR 'mathematical model'/exp OR 'mathematical model' OR 'mathematical models' OR 'systems analysis'/exp OR 'systems analysis' OR 'systems analyses' OR 'systems oriented approach' OR 'systems oriented approaches' OR 'system dynamics analysis' OR 'system dynamics analyses' OR 'systems approach' OR 'systems approaches' OR 'systems thinking'/exp OR 'systems thinking' OR 'agent-based modeling' OR 'agent-based model' OR 'agent based model'/exp OR 'agent based model' OR 'agent based modeling'/exp OR 'agent based modeling' OR 'markov

chain'/exp OR 'markov chain' OR 'markov process'/exp OR 'markov process' OR 'markov processes' OR 'cost-benefit analysis'/exp OR 'cost-benefit analysis' OR 'cost-benefit analyses' OR 'cost benefit analysis'/exp OR 'cost benefit analysis' OR 'cost benefit analyses' OR 'cost-benefit data' OR 'cost benefit data' OR 'cost-utility analysis'/exp OR 'cost-utility analysis' OR 'cost-utility analyses' OR 'cost utility analysis'/exp OR 'cost utility analysis' OR 'cost utility analyses' OR 'economic evaluation'/exp OR 'economic evaluation' OR 'economic evaluations' OR 'cost benefit'/exp OR 'cost benefit' OR 'costs and benefits' OR 'benefits and costs' OR 'cost-effectiveness analysis'/exp OR 'cost-effectiveness analysis' OR 'cost-effectiveness analyses' OR 'economic model'/exp OR 'economic model' OR 'economic models' OR 'individual based model'/exp OR 'individual based model' OR 'individual based modeling' OR 'individual-based model' OR 'individual-based modeling' OR 'microsimulation'/exp OR microsimulation OR 'microsimulation model'/exp OR 'microsimulation model' OR 'microsimulation modeling' OR 'simulation model'/exp OR 'simulation model' OR 'simulation modeling' OR 'cohort model' OR 'cohort modeling' OR 'compartmental model'/exp OR 'compartmental model' OR 'compartmental modeling' OR 'markov model'/exp OR 'markov model' OR 'markov modeling') AND ('health care policy'/exp OR 'health care policy' OR 'health promotion'/exp OR 'health promotion' OR 'diet, healthy'/exp OR 'diet, healthy' OR 'public health'/exp OR 'public health' OR 'health policies' OR 'health policy'/exp OR 'health policy' OR 'nutrition policy'/exp OR 'nutrition policy' OR 'nutrition policies' OR 'food policy'/exp OR 'food policy' OR 'food policies' OR 'nutrition guideline' OR 'nutrition guidelines' OR 'dietary guideline'/exp OR 'dietary guideline' OR 'dietary guidelines' OR 'public health':ti,ab OR 'population health':ti,ab OR 'health benefits':ti,ab OR 'policy scenario' OR 'policy impact' OR 'policy intervention') AND ('sugar-sweetened beverage'/exp OR 'sugar-sweetened beverage' OR ((ssb:ti,ab OR ssbs:ti,ab) AND (beverage*:ti,ab OR drink*:ti,ab OR juic*:ti,ab OR soda*:ti,ab)) OR 'sweet drink' OR 'sweet drinks' OR 'sweet beverage' OR 'sweet beverages' OR 'sweetened drink' OR 'sweetened drinks' OR 'sweetened beverage'/exp OR 'sweetened beverage' OR 'sweetened beverages' OR 'sugary drink' OR 'sugary drinks' OR 'sugary beverage' OR 'sugary beverages' OR 'carbonated beverage'/exp OR 'carbonated beverage' OR 'carbonated beverages'/exp OR 'carbonated beverages' OR 'carbonated drink'/exp OR 'carbonated drink' OR 'carbonated drinks' OR 'soft drink'/exp OR 'soft drink' OR 'soft drinks'/exp OR 'soft drinks' OR soda:ti,ab OR sodas:ti,ab OR 'soda pop' OR 'soda pops' OR cola:ti,ab OR 'coca cola'/exp OR 'coca cola' OR 'flavored water' OR 'flavored waters' OR 'flavoured water' OR 'flavoured waters' OR 'juice'/exp OR juice OR juices OR 'fruit-flavored drink' OR 'fruit-flavored drinks' OR 'fruit-flavoured drink' OR 'fruit-flavoured drinks' OR 'fruit-flavored beverage' OR 'fruit-flavored beverages' OR 'fruit-flavoured beverage' OR 'fruit-flavoured beverages' OR drink:ti,ab OR drinks:ti,ab OR beverage:ti,ab OR beverages:ti,ab)

3 CINAHL

Searched June 25, 2020

351 results

updated search June 10, 2021

35 results ((MH "Life Table method+") OR "life table" OR "life tables" OR life-table OR life-tables OR (MH "Computer Simulation+") OR (MH "Quality-Adjusted Life Years+") OR (MH "Models, Theoretical+") OR (MH "Systems Analysis+") OR (MH "Cost Benefit Analysis+") OR "computer simulation" OR "computer simulations" OR "computer model" OR "computer models" OR "computerized model" OR "computerized models" OR QALY OR QALYs OR "quality-adjusted life year" OR "quality-adjusted life years" OR "quality

adjusted life year" OR "quality adjusted life years" OR "adjusted life year" OR "adjusted life years" OR "healthy years equivalent" OR "healthy years equivalents" OR "theoretical model" OR "theoretical models" OR "mathematical model" OR "mathematical models" OR "systems analysis" OR "systems analyses" OR "systems oriented approach" OR "systems oriented approaches" OR "system dynamics analysis" OR "system dynamics analyses" OR "systems approach" OR "systems approaches" OR "systems thinking" OR "agent-based modeling" OR "agent-based model" OR "agent based model" OR "agent based modeling" OR "markov chain" OR “markov chiains” OR "markov process" OR "markov processes" OR "cost-benefit analysis" OR "cost-benefit analyses" OR "cost benefit analysis" OR "cost benefit analyses" OR "cost-benefit data" OR "cost benefit data" OR "cost-utility analysis" OR "cost-utility analyses" OR "cost utility analysis" OR "cost utility analyses" OR "economic evaluation" OR "economic evaluations" OR "cost benefit" OR "costs and benefits" OR "benefits and costs" OR "cost-effectiveness analysis" OR "cost-effectiveness analyses" OR "economic model" OR "economic models" OR "individual based model" OR "individual based modeling" OR "individual-based model" OR "individual-based modeling" OR microsimulation OR "microsimulation model" OR "microsimulation modeling" OR "simulation model" OR "simulation modeling" OR "cohort model" OR "cohort modeling" OR "compartmental model" OR "compartmental modeling" OR "markov model" OR "markov modeling") AND ((MH "Health Policy+") OR (MH "Nutrition Policy+") OR (MH "Health Promotion+") OR (MH "Public Health+") OR "health policies" OR "health policy" OR "nutrition policy" OR "nutrition policies" OR "food policy" OR "food policies" OR "nutrition guideline" OR "nutrition guidelines" OR "dietary guideline" OR "dietary guidelines" OR TI "public health" OR AB "public health" OR TI "population health" OR AB "population health" OR TI "health benefits" OR AB "health benefits" OR "policy scenario" OR "policy impact" OR "policy intervention") AND ((MH "Carbonated Beverages+") OR (MH “Sweetened Beverages+") OR ((TI ssb OR AB ssb OR TI ssbs OR AB ssbs) AND (TI beverage* OR AB beverage* OR TI drink* OR AB drink* OR TI juic* OR AB juic* OR TI soda* OR AB soda*)) OR "sweet drink" OR "sweet drinks" OR "sweet beverage" OR "sweet beverages" OR "sweetened drink" OR "sweetened drinks" OR "sweetened beverage" OR "sweetened beverages" OR "sugary drink" OR "sugary drinks" OR "sugary beverage" OR "sugary beverages" OR "carbonated beverage" OR "carbonated beverages" OR "carbonated drink" OR "carbonated drinks" OR "soft drink" OR "soft drinks" OR TI soda OR AB soda OR TI sodas OR AB sodas OR soda-pop OR "soda pop" OR "soda pops" OR TI cola OR AB cola OR coca-cola OR "flavored water" OR "flavored waters" OR "flavoured water" OR "flavoured waters" OR juice OR juices OR "fruit-flavored drink" OR "fruit-flavored drinks" OR "fruit-flavoured drink" OR "fruit-flavoured drinks" OR "fruit-flavored beverage" OR "fruit-flavored beverages" OR "fruit-flavoured beverage" OR "fruit-flavoured beverages" OR TI drink OR AB drink OR TI drinks OR AB drinks OR TI beverage OR AB beverage OR TI beverages OR AB beverages)

4 Cochrane Reviews

460 results

Searched June 25, 2020

updated search June 10, 2021

9 results

(life table OR life tables OR life-table OR life-tables OR "Computer Simulation" OR "Quality-Adjusted Life Years" OR "Systems Analysis" OR computer simulation OR computer simulations OR computer model OR computer models OR computerized model OR computerized models OR QALY OR QALYs OR quality-adjusted life year OR quality-adjusted life years OR quality adjusted life year OR quality adjusted life years OR adjusted life year OR adjusted life years OR healthy years equivalent OR healthy years equivalents OR theoretical model OR theoretical models OR mathematical model OR mathematical models OR systems analysis OR systems analyses OR systems oriented approach OR systems oriented approaches OR system dynamics analysis OR system dynamics analyses OR systems approach OR systems approaches OR systems thinking OR agent-based modeling OR agent-based model OR agent based model OR agent based modeling OR markov chain OR markov process OR markov processes OR cost-benefit analysis OR cost-benefit analyses OR cost benefit analysis OR cost benefit analyses OR cost-benefit data OR cost benefit data OR cost-utility analysis OR cost-utility analyses OR cost utility analysis OR cost utility analyses OR economic evaluation OR economic evaluations OR cost benefit OR "costs and benefits" OR "benefits and costs" OR cost-effectiveness analysis OR cost-effectiveness analyses OR economic model OR economic models OR individual based model OR individual based modeling OR individual-based model OR individual-based modeling OR microsimulation OR microsimulation model OR microsimulation modeling OR simulation model OR simulation modeling OR cohort model OR cohort modeling OR compartmental model OR compartmental modeling OR markov model OR markov modeling)

AND (health policies OR health policy OR nutrition policy OR nutrition policies OR food policy OR food policies OR nutrition guideline OR nutrition guidelines OR dietary guideline OR dietary guidelines OR public health OR population health OR health benefits OR policy scenario OR policy impact OR policy intervention)

AND (sweet drink OR sweet drinks OR sweet beverage OR sweet beverages OR sweetened drink OR sweetened drinks OR sweetened beverage OR sweetened beverages OR sugary drink OR sugary drinks OR sugary beverage OR sugary beverages OR carbonated beverage OR carbonated beverages OR carbonated drink OR carbonated drinks OR soft drink OR soft drinks OR soda OR sodas OR soda-pop OR soda pop OR soda pops OR cola OR coca-cola OR flavored water OR flavored waters OR flavoured water OR flavoured waters OR juice OR juices OR fruit-flavored drink OR fruit-flavored drinks OR fruit-flavoured drink OR fruit-flavoured drinks OR fruit-flavored beverage OR fruit-flavored beverages OR fruit-flavoured beverage OR fruit-flavoured beverages OR drink OR drinks OR beverage OR beverages OR ((ssb OR ssbs) AND (beverage* OR drink* OR juic* OR soda*)) OR sweet drink OR sweet drinks OR sweet beverage OR sweet beverages OR sweetened drink OR sweetened drinks OR sweetened beverage OR sweetened beverages OR sugary drink OR sugary drinks OR sugary beverage OR sugary beverages OR carbonated beverage OR carbonated beverages OR carbonated drink OR carbonated drinks OR soft drink OR soft drinks OR soda OR sodas OR soda-pop OR soda pop OR soda pops OR cola OR coca-cola OR flavored water OR flavored waters OR flavoured water OR flavoured waters OR juice OR juices OR fruit-flavored drink OR fruit-flavored drinks OR fruit-flavoured drink OR fruit-flavoured drinks OR fruit-flavored beverage OR fruit-flavored beverages OR fruit-flavoured beverage OR fruit-flavoured beverages OR drink OR drinks OR beverage OR beverages)

5 Communication and Mass Media Complete

3 results

searched June 25, 2020

updated search June 10, 2021

0 new results

((life table OR life tables OR life-table OR life-tables OR "Computer Simulation" OR "Quality-Adjusted Life Years" OR "Systems Analysis" OR computer simulation OR computer simulations OR computer model OR computer models OR computerized model OR computerized models OR QALY OR QALYs OR quality-adjusted life year OR quality-adjusted life years OR quality adjusted life year OR quality adjusted life years OR adjusted life year OR adjusted life years OR healthy years equivalent OR healthy years equivalents OR theoretical model OR theoretical models OR mathematical model OR mathematical models OR systems analysis OR systems analyses OR systems oriented approach OR systems oriented approaches OR system dynamics analysis OR system dynamics analyses OR systems approach OR systems approaches OR systems thinking OR agent-based modeling OR agent-based model OR agent based model OR agent based modeling OR markov chain OR markov process OR markov processes OR cost-benefit analysis OR cost-benefit analyses OR cost benefit analysis OR cost benefit analyses OR cost-benefit data OR cost benefit data OR cost-utility analysis OR cost-utility analyses OR cost utility analysis OR cost utility analyses OR economic evaluation OR economic evaluations OR cost benefit OR "costs and benefits" OR "benefits and costs" OR cost-effectiveness analysis OR cost-effectiveness analyses OR economic model OR economic models OR individual based model OR individual based modeling OR individual-based model OR individual-based modeling OR microsimulation OR microsimulation model OR microsimulation modeling OR simulation model OR simulation modeling OR cohort model OR cohort modeling OR compartmental model OR compartmental modeling OR markov model OR markov modeling)) AND ((health policies OR health policy OR nutrition policy OR nutrition policies OR food policy OR food policies OR nutrition guideline OR nutrition guidelines OR dietary guideline OR dietary guidelines OR public health OR population health OR health benefits OR policy scenario OR policy impact OR policy intervention)) AND (((ssb OR ssbs) AND (beverage* OR drink* OR juic* OR soda*)) OR sweet drink OR sweet drinks OR sweet beverage OR sweet beverages OR sweetened drink OR sweetened drinks OR sweetened beverage OR sweetened beverages OR sugary drink OR sugary drinks OR sugary beverage OR sugary beverages OR carbonated beverage OR carbonated beverages OR carbonated drink OR carbonated drinks OR soft drink OR soft drinks OR soda OR sodas OR soda-pop OR soda pop OR soda pops OR cola OR coca-cola OR flavored water OR flavored waters OR flavoured water OR flavoured waters OR juice OR juices OR fruit-flavored drink OR fruit-flavored drinks OR fruit-flavoured drink OR fruit-flavoured drinks OR fruit-flavored beverage OR fruit-flavored beverages OR fruit-flavoured beverage OR fruit-flavoured beverages OR drink OR drinks OR beverage OR beverages))

6 PsycInfo

1184 results

searched June 25, 2020

updated search June 10, 2021

42 new results

(("Life Tables" OR "life table" OR DE "Computer Simulation" OR "Quality-Adjusted Life Years" OR DE "Systems Analysis" OR DE "Markov Chains" OR DE "Costs and Cost Analysis" OR "computer simulation" OR "computer simulations" OR "computer model" OR "computer models" OR "computerized model" OR "computerized models" OR QALY OR QALYs OR "quality-adjusted life year" OR "quality-adjusted life years" OR "quality adjusted life year" OR "quality adjusted life years" OR "adjusted life year" OR "adjusted life years" OR "healthy years equivalent" OR "healthy years equivalents" OR "theoretical model" OR "theoretical models" OR "mathematical model" OR "mathematical models" OR "systems analysis" OR "systems analyses" OR "systems oriented approach" OR "systems oriented approaches" OR "system dynamics analysis" OR "system dynamics analyses" OR "systems approach" OR "systems approaches" OR "systems thinking" OR "agent-based modeling" OR "agent-based model" OR "agent based model" OR "agent based modeling" OR "markov chain" OR "markov process" OR "markov processes" OR "cost-benefit analysis" OR "cost-benefit analyses" OR "cost benefit analysis" OR "cost benefit analyses" OR "cost-benefit data" OR "cost benefit data" OR "cost-utility analysis" OR "cost-utility analyses" OR "cost utility analysis" OR "cost utility analyses" OR ""”systems analysis” OR economic evaluation" OR "economic evaluations" OR "cost benefit" OR "costs and benefits" OR "benefits and costs" OR "cost-effectiveness analysis" OR "cost-effectiveness analyses" OR "economic model" OR "economic models" OR "individual based model" OR "individual based modeling" OR "individual-based model" OR "individual-based modeling" OR microsimulation OR "microsimulation model" OR "microsimulation modeling" OR "simulation model" OR "simulation modeling" OR "cohort model" OR "cohort modeling" OR "compartmental model" OR "compartmental modeling" OR "markov model" OR "markov modeling")) AND ((DE "Health Promotion" OR “health promotion” OR DE "Public Health OR "health policies" OR "health policy" OR "nutrition policy" OR "nutrition policies" OR "food policy" OR "food policies" OR "nutrition guideline" OR "nutrition guidelines" OR "dietary guideline" OR "dietary guidelines" OR "public health" OR "population health" OR "health benefits" OR "policy scenario" OR "policy impact" OR "policy intervention")) AND (("Carbonated Beverages" OR "Sugar-Sweetened Beverages" OR ((TI ssb OR AB ssb OR TI ssbs OR AB ssbs) AND (TI beverage* OR AB beverage* OR TI drink* OR AB drink* OR TI juic* OR AB juic* OR TI soda* OR AB soda*)) OR "sweet drink" OR "sweet drinks" OR "sweet beverage" OR "sweet beverages" OR "sweetened drink" OR "sweetened drinks" OR "sweetened beverage" OR "sweetened beverages" OR "sugary drink" OR "sugary drinks" OR "sugary beverage" OR "sugary beverages" OR "carbonated beverage" OR "carbonated beverages" OR "carbonated drink" OR "carbonated drinks" OR "soft drink" OR "soft drinks" OR TI soda OR AB sodas OR TI sodas OR AB Sodas OR soda-pop OR "soda pop" OR "soda pops" OR TI cola OR AB cola OR TI Colas OR AB colas OR coca-cola OR "flavored water" OR "flavored waters" OR "flavoured water" OR "flavoured waters" OR juice OR juices OR "fruit-flavored drink" OR "fruit-flavored drinks" OR "fruit-flavoured drink" OR "fruit-flavoured drinks" OR "fruit-flavored beverage" OR "fruit-flavored beverages" OR "fruit-flavoured beverage" OR "fruit-flavoured beverages"))

7 Scopus

598 results

Searched June 25, 2020

updated search June 10, 2021

29 results

TITLE-ABS-KEY ("life table" OR "life tables" OR "life-table" OR "life-tables" OR "Computer Simulation" OR "Quality-Adjusted Life Years" OR "theoretical model" OR "theoretical models" OR "Systems Analysis" OR "Cost-Benefit Analysis" OR "Economic model" OR "economic models" OR "computer simulation" OR "computer simulations" OR "computer model" OR "computer models" OR "computerized model" OR "computerized models" OR "QALY" OR "QALYs" OR "quality-adjusted life year" OR "quality-adjusted life years" OR "quality adjusted life year" OR "quality adjusted life years" OR "adjusted life year" OR "adjusted life years" OR "healthy years equivalent" OR "healthy years equivalents" OR "theoretical model" OR "theoretical models" OR "mathematical model" OR "mathematical models" OR "systems analysis" OR "systems analyses" OR "systems oriented approach" OR "systems oriented approaches" OR "system dynamics analysis" OR "system dynamics analyses" OR "systems approach" OR "systems approaches" OR "systems thinking" OR "agent-based modeling" OR "agent-based model" OR "agent based model" OR "agent based modeling" OR "markov chain" OR "markov process" OR "markov processes" OR "cost-benefit analysis" OR "cost-benefit analyses" OR "cost benefit analysis" OR "cost benefit analyses" OR "cost-benefit data" OR "cost benefit data" OR "cost-utility analysis" OR "cost-utility analyses" OR "cost utility analysis" OR "cost utility analyses" OR "economic evaluation" OR "economic evaluations" OR "cost benefit" OR "costs and benefits" OR "benefits and costs" OR "cost-effectiveness analysis" OR "cost-effectiveness analyses" OR "economic model" OR "economic models" OR "individual based model" OR "individual based modeling" OR "individual-based model" OR "individual-based modeling" OR "microsimulation" OR "microsimulation model" OR "microsimulation modeling" OR "simulation model" OR "simulation modeling" OR "cohort model" OR "cohort modeling" OR "compartmental model" OR "compartmental modeling" OR "markov model" OR "markov modeling") AND TITLE-ABS-KEY ("Health Policy" OR "Nutrition Policy" OR "Health Promotion" OR "healthy diet" OR "healthy diets" OR "Public Health" OR "health policies" OR "health policy" OR "nutrition policy" OR "nutrition policies" OR "food policy" OR "food policies" OR "nutrition guideline" OR "nutrition guidelines" OR "dietary guideline" OR "dietary guidelines" OR "population health" OR "health benefits" OR "policy scenario*" OR "policy impact" OR "policy intervention") AND TITLE-ABS-KEY ("sweet drink" OR "sweet drinks" OR "sweet beverage" OR "sweet beverages" OR "sweetened drink" OR "sweetened drinks" OR "sweetened beverage" OR "sweetened beverages" OR "sugary drink" OR "sugary drinks" OR "sugary beverage" OR "sugary beverages" OR "carbonated beverage" OR "carbonated beverages" OR "carbonated drink" OR "carbonated drinks" OR "soft drink" OR "soft drinks" OR "soda" OR "sodas" OR "soda-pop" OR "soda pop" OR "soda pops" OR ("cola") OR "coca-cola" OR "flavored water" OR "flavored waters" OR "flavoured water" OR "flavoured waters" OR "juice" OR "juices" OR "fruit-flavored drink" OR "fruit-flavored drinks" OR "fruit-flavoured drink" OR "fruit-flavoured drinks" OR "fruit-flavored beverage" OR "fruit-flavored beverages" OR "fruit-flavoured beverage*" OR (("ssb" OR "ssbs") AND ("beverage*" OR "drink*" OR "juic*" OR "soda*")))
