# Supplementary material for: Simulation models of sugary drink policies: A scoping review
Source: PLoS One. 2022 Oct 3;17(10):e0275270. doi: 10.1371/journal.pone.0275270 (PMC9529101; doi:10.1371/journal.pone.0275270)
Supplement: S1 Table — (DOCX) [file pone.0275270.s004.docx]

S1 Table. Article specific information (n=61)
Interactive table can be found at <https://natsmith.shinyapps.io/Article-Information/>

| Article | SSB Policies | Non-SSB Policies Simulated | Country | Selected Characteristics of Population | Health Outcomes | Method | Primary Time Horizon(s) | Used Existing Model | Visual of Model | Conducted Sensitivity or Uncertainty Analyses | Provided Data or Code | Involved Stakeholders |
| --- | --- | --- | --- | --- | --- | --- | --- | --- | --- | --- | --- | --- |
| Barrientos-Gutierrez, 2017  (PLoS One) | Volumetric Tax | No | Mexico | Age; Sex or gender; SES measure | Weight related; Diabetes | Other or not stated | 10 years | No | No | Yes | Yes | No |
| Basto-Abreu, 2018  (PLoS Med) | Reformulation | No | Mexico | Age; Sex or gender; SES measure | Weight related | Other or not stated | Other | No | No | Yes | Yes | No |
| Basto-Abreu, 2019  (Health Aff (Millwood)) | Volumetric Tax | No | Mexico | Age; Sex or gender | Weight related; Diabetes; Cardiovascular disease; Cancer | Markov cohort modeling | 10 years | Yes | No | Yes | No | No |
| Basu, 2013  (Med Decis Making) | Volumetric Tax; Purchasing Bans | Yes | US | Age; Sex or gender; Race, ethnicity, nativity, or similar; Income; SNAP status | Weight related; Diabetes; Cardiovascular disease | Microsimulation | 10 years | No | Yes | Yes | No | No |
| Basu, 2014  (Am J Public Health) | Volumetric Tax | Yes | US | Age; Sex or gender; Race, ethnicity, nativity, or similar | Weight related; Diabetes | Agent-based modeling | 20 years | No | Yes | Yes | No | No |
| Basu, 2014  (Health Affairs) | Purchasing Bans | Yes | US | Age; Sex or gender; Race, ethnicity, nativity, or similar; Income | Weight related; Diabetes | Microsimulation | 10 years | Yes | No | Yes | No | No |
| Basu, 2014  (PLoS Med) | Ad valorem Tax | No | India | Age; Sex or gender; Income | Weight related; Diabetes | Microsimulation | 10 years | No | Yes | Yes | No | No |
| Basu, 2020  (Health Aff (Millwood)) | Purchasing Bans | No | US (Employee populations of California health care organizations) | Age; Sex or gender; Race, ethnicity, nativity, or similar | Weight related; Diabetes; Cardiovascular disease; Dental caries; Kidney Disease | Microsimulation | 10 years; Lifetime | No | No | Yes | Yes | No |
| Bourke, 2018  (BMJ Glob Health) | Volumetric Tax | No | Indonesia | Age; Sex or gender; Income | Weight related; Diabetes; Cardiovascular disease | Life-table modeling | Lifetime | Yes | No | Yes | No | No |
| Briggs, 2013  (BMC Public Health) | Ad valorem Tax | No | Ireland | Age; Sex or gender; Income | Weight related | Comparative risk assessment | Unclear | Yes | Yes | Yes | No | Yes |
| Briggs, 2013  (BMJ (Online)) | Ad valorem Tax | No | UK | Age; Sex or gender; Income | Weight related | Comparative risk assessment | Unclear | Yes | Yes | Yes | No | No |
| Briggs, 2017  (Lancet Public Health) | Tiered Sugar Tax | No | UK | Age; Sex or gender | Weight related; Diabetes; Dental caries | Comparative risk assessment | Unclear | No | Yes | Yes | No | No |
| Choi, 2021  (Am J Prev Med) | Purchasing Bans | No | US | Age; Sex or gender; Race, ethnicity, nativity, or similar; Income; SNAP status | Weight related; Dental caries | Microsimulation | 10 years | No | Yes | Yes | No | No |
| Cleghorn, 2019  (Prev Med) | Portion Size Restriction | No | New Zealand | Age; Sex or gender; Race, ethnicity, nativity, or similar | Weight related; Diabetes; Cardiovascular disease; Cancer; Osteoarthritis | Life-table modeling | Lifetime | Yes | No | Yes | No | No |
| Cobiac, 2017  (PLoS Med) | Volumetric Tax | Yes | Australia | Age; Sex or gender | Weight related; Diabetes; Cardiovascular disease; Cancer | Life-table modeling | Lifetime | Yes | No | Yes | No | No |
| Collins, 2015  (PLoS One) | Ad valorem Tax | No | England | Age; Sex or gender | Diabetes; Cardiovascular disease; Cancer | Other or not stated | Unclear | No | Yes | Yes | Yes | Yes |
| Crino, 2017  (Nutrients) | Portion Size Restriction; Reformulation | No | Australia | Age; Sex or gender | Weight related; Diabetes; Cardiovascular disease; Cancer; Osteoarthritis | Life-table modeling | Lifetime | Yes | Yes | Yes | No | No |
| Dharmasena, 2012  (Health Econ) | Ad valorem Tax | No | US |  | Weight related | Other or not stated | Unclear | No | No | No | No | No |
| Du, 2020  (JNCI Cancer Spectr) | Volumetric Tax | No | US | Age; Sex or gender; Race, ethnicity, nativity, or similar; Income | Weight related; Cancer | Markov cohort modeling | Lifetime | Yes | No | Yes | No | No |
| Goiana-da-Silva, 2020  (PLoS Med) | Tiered Sugar Tax | No | Portugal | Age | Weight related | Other or not stated | Unclear | No | No | No | No | No |
| Gortmaker, 2015  (Health Aff (Millwood)) | Volumetric Tax | No | US | Age; Sex or gender | Weight related | Microsimulation | 10 years | Yes | No | Yes | No | Yes |
| Grummon, 2019  (Am J Prev Med) | Warning Label | No | US | Age; Sex or gender; Race, ethnicity, nativity, or similar; Education; Income | Weight related | Microsimulation | Other | No | Yes | Yes | No | No |
| Grummon, 2019  (Science) | Volumetric Tax; Nutrient Tax | No | US |  | Weight related; Diabetes | Other or not stated | Unclear | No | No | No | No | No |
| Hangoma, 2020  (BMJ Glob Health) | Ad valorem Tax | No | Zambia | Age; Sex or gender | Weight related | Life-table modeling | Other | No | Yes | Yes | No | Yes |
| Huse, 2020  (Int J Obes (Lond)) | Restrictions on Price Promotions | No | Australia | Age; Sex or gender | Weight related; Diabetes; Cardiovascular disease; Cancer; Osteoarthritis | Life-table modeling | Lifetime | Yes | Yes | Yes | No | No |
| Jevdjevic, 2019  (Public Health) | Ad valorem Tax | No | Netherlands | Age; Sex or gender | Dental caries | Markov cohort modeling | Lifetime | No | Yes | Yes | No | No |
| Kao, 2020  (Econ Hum Biol) | Ad valorem Tax | No | Canada | Age; Sex or gender; Income | Weight related; Diabetes; Cardiovascular disease; Cancer; Osteoarthritis; Kidney Disease | Life-table modeling | Lifetime | Yes | Yes | Yes | No | No |
| Kristensen, 2014  (Am J Prev Med) | Volumetric Tax | Yes | US | Age; Sex or gender; Race, ethnicity, nativity, or similar | Weight related | Microsimulation | 20 years | No | No | Yes | No | No |
| Lal, 2017  (PLoS Med) | Ad valorem Tax | No | Australia | Age; Sex or gender; SES measure | Weight related; Diabetes; Cardiovascular disease; Cancer; Osteoarthritis | Life-table modeling | Lifetime | Yes | Yes | Yes | Yes | No |
| Lee, 2018  (Am J Prev Med) | Warning Label | No | US (Baltimore; San Francisco; Philadelphia) | Age; Sex or gender; Race, ethnicity, nativity, or similar | Weight related | Agent-based modeling | Other | No | Yes | Yes | No | No |
| Lee, 2020  (Circulation) | Volumetric Tax; Tiered Sugar Tax; Nutrient Tax | No | US | Age; Sex or gender; Race, ethnicity, nativity, or similar; Income | Weight related; Diabetes; Cardiovascular disease | Microsimulation | 10 years; Lifetime | Yes | Yes | Yes | No | No |
| Lin, 2011  (Econ Hum Biol) | Ad valorem Tax | No | US | Age; Sex or gender; Income | Weight related | Other or not stated | 10 years | No | No | Yes | No | No |
| Liu, 2016  (Journal of the Operational Research Society) | Volumetric Tax | No | US | Age; Sex or gender | Weight related | System dynamics modeling | Unclear | No | Yes | Yes | No | Yes |
| Pearson-Stuttard, 2017  (PLoS Med) | Ad valorem Tax | Yes | US | Age; Sex or gender; SNAP status | Cardiovascular disease | Other or not stated | Other | Yes | No | Yes | No | No |
| Long, 2015  (Am J Prev Med) | Volumetric Tax | No | US | Age; Sex or gender | Weight related; Diabetes; Cardiovascular disease; Cancer; Osteoarthritis | Markov cohort modeling | 10 years | Yes | No | Yes | No | Yes |
| Long, 2019  (J Nutr Educ Behav) | Volumetric Tax; Purchasing Bans | No | US (Maine) | Age; Sex or gender; SNAP status | Weight related | Microsimulation | 10 years | Yes | No | Yes | No | Yes |
| Ma, 2016  (Lancet Diabetes Endocrinol) | Reformulation | No | UK | Age; Sex or gender; Income | Weight related; Diabetes | Other or not stated | Other | No | Yes | Yes | No | No |
| Manyema, 2014  (PLoS One) | Ad valorem Tax | No | South Africa | Age; Sex or gender | Weight related | Other or not stated | Unclear | No | Yes | Yes | No | No |
| Manyema, 2015  (PLoS One) | Ad valorem Tax | No | South Africa | Age; Sex or gender | Weight related; Diabetes | Life-table modeling | 20 years; Lifetime | No | Yes | Yes | No | No |
| Manyema, 2016  (BMC Public Health) | Ad valorem Tax | No | South Africa | Age; Sex or gender | Weight related; Cardiovascular disease | Life-table modeling | 20 years | No | Yes | Yes | No | No |
| Mekonnen, 2013  (PLoS One) | Volumetric Tax | No | US (California) | Age; Sex or gender; Race, ethnicity, nativity, or similar; Income | Weight related; Diabetes; Cardiovascular disease | Other or not stated | 10 years | Yes | Yes | Yes | No | No |
| Nomaguchi, 2017  (Health Policy) | Ad valorem Tax | No | Australia | Age; Sex or gender | Weight related; Diabetes; Cardiovascular disease; Cancer; Osteoarthritis | Life-table modeling | Other | Yes | Yes | Yes | No | No |
| Peñalvo, 2017  (BMC Med) | Ad valorem Tax | Yes | US | Age; Sex or gender; Education | Diabetes; Cardiovascular disease | Comparative risk assessment | Unclear | No | No | Yes | No | No |
| Phonsuk, 2021  (PLoS One) | Ad valorem Tax | No | Thailand | Age; Sex or gender | Weight related | Other or not stated | Unclear | No | Yes | Yes | No | No |
| Rosettie, 2018  (PLoS One) | Restrictions on Availability | Yes | US | Age; Sex or gender; Race, ethnicity, nativity, or similar | Weight related; Diabetes; Cardiovascular disease | Comparative risk assessment | Unclear | No | No | Yes | No | No |
| Ruff, 2015  (Ann Epidemiol) | Nutrient Tax | No | US (New York City) | Age; Sex or gender | Weight related | Other or not stated | 10 years | No | No | No | No | No |
| Sánchez-Romero, 2016  (PLoS Med) | Ad valorem Tax | No | Mexico | Age; Sex or gender | Weight related; Diabetes; Cardiovascular disease | Markov cohort modeling | 10 years | Yes | No | Yes | No | No |
| Saxena, 2019  (Bull World Health Organ) | Volumetric Tax | No | Philippines | Age; Sex or gender; Income | Weight related; Diabetes; Cardiovascular disease | Other or not stated | 20 years | Yes | No | Yes | Yes | No |
| Schwendicke, 2016  (J Dent Res) | Ad valorem Tax | No | Germany | Age; Sex or gender; Income | Dental caries | Microsimulation | 10 years | No | Yes | Yes | No | No |
| Schwendicke, 2017  (BMC Public Health) | Ad valorem Tax | No | Germany | Age; Sex or gender; Income | Weight related | Microsimulation | Other | Yes | No | Yes | No | No |
| Segovia, 2020  (PLoS One) | Volumetric Tax; Ad valorem Tax | No | Ecuador | Age; Sex or gender; Education; Income | Weight related | Other or not stated | Unclear | No | No | No | No | No |
| Sharma, 2014  (Health Econ) | Volumetric Tax; Ad valorem Tax | No | Australia | Age; Sex or gender; Income | Weight related | Other or not stated | Unclear | No | No | Yes | No | No |
| Sowa, 2019  (Eur J Public Health) | Ad valorem Tax | No | Australia | Age; Sex or gender | Dental caries | Markov cohort modeling | 10 years | No | Yes | Yes | No | No |
| Stacey, 2018  (BMJ Glob Health) | Volumetric Tax | Yes | South Africa | Age; Sex or gender | Weight related | Life-table modeling | Other | Yes | Yes | Yes | No | Yes |
| Summan, 2020  (BMJ Glob Health) | Ad valorem Tax | Yes | Global | Age; Sex or gender | Weight related | Life-table modeling | Other | No | Yes | Yes | No | No |
| Torres-Álvarez, 2020  (Pediatr Obes) | Volumetric Tax | No | Mexico | Age; Sex or gender | Weight related | Other or not stated | Other | No | No | Yes | Yes | No |
| Urwannachotima, 2020  (BMC Oral Health) | Tiered Sugar Tax | No | Thailand | Age; Sex or gender; Income | Dental caries | System dynamics modeling | Other | No | Yes | Yes | No | Yes |
| Vecino-Ortiz, 2018  (Social Science and Medicine) | Volumetric Tax | No | Colombia | Age; Sex or gender; Education; SES measure | Weight related | Comparative risk assessment | Other | No | No | Yes | No | No |
| Veerman, 2016  (PLoS One) | Ad valorem Tax | No | Australia | Age; Sex or gender | Weight related; Diabetes; Cardiovascular disease; Cancer; Osteoarthritis | Life-table modeling | Lifetime | Yes | No | Yes | Yes | No |
| Wang, 2012  (Health Affairs) | Volumetric Tax | No | US | Age; Sex or gender | Weight related; Diabetes; Cardiovascular disease | Life-table modeling | 10 years | Yes | No | Yes | No | No |
| Wilde, 2019  (Am J Public Health) | Volumetric Tax | No | US | Age; Sex or gender; Race, ethnicity, nativity, or similar; Income | Weight related; Diabetes; Cardiovascular disease | Microsimulation | Lifetime | Yes | No | Yes | No | No |
