# Supplementary material for: Simulation models of sugary drink policies: A scoping review
Source: PLoS One. 2022 Oct 3;17(10):e0275270. doi: 10.1371/journal.pone.0275270 (PMC9529101; doi:10.1371/journal.pone.0275270)
Supplement: Attachment — Submitted filename: ssbSimReview_response-memo_submission.docx [file pone.0275270.s005.docx]

**Introduction to Response to Reviewers**

We are pleased to submit our revised manuscript “Simulation models of sugary drink policies: A scoping review” for potential publication in PLOS ONE. We thank the reviewers for taking the time to review our manuscript thoughtfully and constructively. Comments from reviewers are included below along with our responses in text below each, noting page numbers where changes were made to the manuscript as appropriate. We number each reviewer comment noting reviewer number followed by comment number (e.g., Reviewer 2 comment 3 is R2.3). We have indicated modifications made to the manuscript using track changes, with substantive changes highlighted (we do not highlight formatting changes). A clean copy has also been submitted.

We have also made the requested formatting changes, including modifications to headers, citation style, and supplementary file naming. We have reviewed the reference list; we do not cite any articles that have been retracted and note additions to the reference list in our responses to reviewers below.

**Reviewer 1**

1) In the search terms, 'modeling' may also be spelled as 'modelling' with two 'l's. You may want to make greater use of the asterisk '*' for expanding search terms in future.

   **R1.1**. Thank you for this comment. We had originally searched with the term modelling, and we did not retrieve any additional articles. If we repeat these or similar searches in the future, we will consider modifying the search strategy to include asterisks to make this more explicit.

2) On page 6, "we included an article known to our team that was not picked up by search terms because it did not have an abstract". It would be interesting to know why this wasn't found by keyword matches in the title, and if you considered amending your search strategy. Perhaps there are other articles without abstracts that will have been excluded?

   **R1.2**. Thank you for this question. The article’s title was “Designing better sugary drink taxes;” the search terms did not pick up this paper because there was no mention of simulation modeling in the title. We considered amending the search strategy to not require terms related to modeling, as such a strategy might have picked up this article. However, the current strategy returned a large number of records (~5,000 after removing duplicates, to yield a total of 60 included studies), so we opted against a broader search for feasibility reasons. Additionally, it is uncommon for articles to lack abstracts, so we do not think the search strategy used missed many articles for this reason.
3) Also this article is referenced on page 7 (80) but perhaps reference it on page 6 as well, where you mention it for the first time.

   **R1.3**. Revised as suggested; this reference is now reference #19.
4) Tangent: I have been working on my own scoping review recently and I constructed a 'test set' of papers and tested my search strategy against this set to see if it retrieved all the papers I wanted. This might be an interesting technique for you in future reviews.

   **R1.4**. Thank you for this comment – we also used this strategy, though informally. We noted eight papers that had been published between 2015 and 2019 that covered a variety of sugary drink policies and modeling methods and made sure that each was picked up in at least one of our database searches.
5) Thank you for publishing your code and your extracted data! :) This is very useful for other people who want to dig into your results more.

   **R1.5**. Thank you for this comment.
6) I would also suggest publishing the original results of your database searches and including a column to indicate at which stage each result was excluded. This would allow readers to gain more insight into your screening process, perform additional checks, and mine your results for more specific or slightly different literature / research questions. I don't know if you can export this data from Covidence, but it might be a quick win if possible. I would also include data on exclusions at the full text/eligibility stage as well.

   **R1.6**. Thank you for this suggestion. We are not able to export what articles were excluded at the title/abstract screening stage from Covidence but have included a new supplementary file that includes the 82 articles we excluded during full text screening along with exclusion reasons (S3 File).
7) The interactive summary table on shinyapps.io is a really nice touch! :)

   **R1.7**. Thank you.
8) Page 8 uses the phrase "ad valorem tax" which is not a term I have come across. Perhaps a short parenthetical to explain what these are like "(value added taxes which are set at a fixed percentage of the final sale price)" would be good.

   **R1.8**. Thank you for bringing this to our attention – we have added a parenthetical on page 8. The sentence now reads: “To fully characterize taxes, we extracted information on both the tax rate (e.g., *ad valorem*/percentage-based, such as 20%, or unit-based, such as 1 cent-per-fluid-ounce) and how that tax rate would be implemented (i.e., excise, sales, other, unclear), based on the exact language used in the article.” (page 7)
9) In table 1, it is not clear to me what "quality outcome" means. Please can you add a description or clarify? Does this mean "(health related) quality of life"?

   **R1.9**. We have modified this row to read ‘Quality of life outcome’ and provided an example for both quality of life outcomes and economic outcomes in the table notes (page 10).
10) Thank you for reporting on the publication of code and data in these models, it is something I care deeply about! What a shame only 8 papers published their code!

    **R1.10**. We also think this is very important and are glad you think so as well.
11) The stakeholder involvement comment is also very interesting!

    **R1.11**. Thank you.
12) On page 13 you mention the increase in publication over time. Perhaps a line chart of cumulative published studies over time" would be interesting?

    **R1.12**. Although we want to draw attention to the increased focus on simulation modeling of these policies, we are also aware that in general trends in number of publications have been rising, and so did not want to put too much emphasis on the trend for modeling studies specifically. The Shiny app also makes it feasible to see specific numbers of articles per year for those who are interested.
13) On page 13 you state "different tax rates – such as volume-based vs. sugar-based taxes – can have markedly different impacts on SSB consumption and health outcomes, even if they are economically equivalent" which points to other drivers of change apart from price signalling. This sounds very counterintuitive, and it's not immediately clear to me what these other drivers would be (perhaps reformulations or repackaging?). Please could you add an example to this paragraph to show how two different types of tax, at the same equivalent rate, could lead to different consumption / health outcomes?

    **R1.13**. We agree that signaling could be one non-price mechanism through which taxes affect purchases. However, we speculate that volume- and sugar-based taxes would have similar signaling effects. Instead, the reason that sugar-based taxes are expected to have larger impacts than volume-based taxes is because sugar-based taxes will tend to lead to larger price increases for higher-sugar vs. lower-sugar SSBs (because the tax is on sugar content), while volume-based taxes lead to similar price increases for all SSBs, regardless of sugar content (because the tax is on volume of beverage sold). Thus, sugar-based taxes could create incentives for consumers to switch from higher- to lower-sugar SSBs, while volume-based taxes would not. Our initial submission used the term ‘economically equivalent’ because these differential incentives could occur even if the two tax types lead to the same average price increase. However, we have removed this term from the text explanation as we think it detracts from the main message about design being important. We have revised to briefly explain this mechanism in the Discussion:

    “This could be problematic because some tax designs can have markedly different impacts on SSB consumption and health outcomes even when they raise prices by the same amount [19,51,96]. For example, one study found that taxing sugar content instead of beverage volume would increase the public health benefits of an SSB tax by 30% because sugar-based taxes could create price incentives for consumers to substitute from higher- to lower-sugar SSBs, while volumetric taxes would not [19].” (page 13).
14) Throughout the article you have sprinkled in critical reflections on the literature you have reviewed, and this is really fantastic, it makes the paper much more informative and interesting to read than just a 'simple' review. :)

    **R1.14**. Thank you for this comment.
15) On page 14 you discuss "pass-through rates" but again I'm not sure what this means. Please could you add a short parenthetical?

    **R1.15**. We apologize for not defining this term clearly – we have now defined pass through earlier in the text where we discuss how tax impacts on SSB consumption were simulated (page 8):

    “Studies generally simulated impacts on consumption by first estimating changes in SSB prices under tax policies, using assumptions about baseline prices of SSBs and the percent of tax passed through (i.e., the amount of tax that the taxed entity ‘passes through’ to consumers via price increases). Changes in prices were then translated to changes in consumption using price elasticities (which quantify the percent change in consumption for a percentage change in price).”
16) On page 15 you discuss some of the benefits of modelling multiple policy options in a single framework. You may also want to mention the needs of policy-makers to evaluate multiple potential options and any negative and spillover effects, and that by presenting evidence on multiple options, researchers can make their outputs more useful to policy-makers and improve the uptake of their evidence into policy.

    **R1.16**. We appreciate this comment and have added your feedback into this paragraph on the bottom of page 15 – top of page 16:

    “Most models we reviewed focused on one policy. An important next step will be for researchers to simulate multiple policy options within one modeling framework to compare policy effectiveness, and possibly expand into comparing policy options with other types of public health action such as community-based programs or interventions. Comparative information can help policymakers weighing multiple paths forward identify tradeoffs given potential limited resources for implementation and limited political capital – potentially making research more useful to policymakers and increasing its use in policy decision making. [paragraph continues]”
17) Thank you also for you emphasis on health equity and the need to examine different (intersectional) population cohorts. Again this is something policy-makers want to know, and it's also such an important part of any health policy research!

    **R1.17**. Thank you!
18) Overall an enjoyable and informative paper which has provided a service to the research and policy communities by drawing together the extant literature! :)

    **R1.18**. Thank you!

**Reviewer #2**

1. I found this article pleasant to read and informative. I have no major comments or concerns, although I do welcome greater clarity and emphasis through summary – perhaps a table – surrounding each study’s causal focus, quantification of potential biases, and mitigation of these potential biases within implementation for each simulation study.

   This is covered comprehensively within the text but adding some explicit causal inference terminology and providing an overall summary might be quite a powerful message. It is because I worry that most scientific studies (whether engaging in simulation or not) seek causal understanding yet do not always explicitly state this; and, whether they do or not, most studies undertake insufficient (or no) evaluation of the robustness of their findings (beyond the usual caveats stated about study limitations in the discussion).

   I do not suggest this to be onerous, as the information is mostly there already, but I would welcome clarity on these issues through a summary of all studies with respect to: which were explicit in their pursuit of causal inference, which stated their causal estimand(s), and the extent to which each sought to assess or quantify robustness of study estimates (e.g., through sensitivity analyses or through any other form of quantitative bias analysis).

   **R2.1**. Thank you for this comment. We have made several revisions to address this comment about the causal focus, quantification of biases, and mitigation of biases:

First, we have added a column denoting whether the study included sensitivity or uncertainty analyses to the online shiny app table and the appendix table.

Second, we considered doing a more in-depth exploration of the extent of sensitivity and uncertainty analyses used by included studies. However, during initial extractions we found that the language used by authors varied widely (e.g., sensitivity vs. uncertainty analyses), and it was often unclear what parameters were included in probabilistic analyses, making it difficult to synthesize these analyses beyond what is currently reported in the manuscript. Instead, we have revised the discussion of sensitivity and uncertainty analyses to note the varied language used to describe sensitivity/uncertainty analyses (page 15):

“…and nearly all reported some form of sensitivity or uncertainty analyses, though the descriptions of such analyses and language used varied widely.”

We also discuss the need to further standardize language used to describe sensitivity and uncertainty analyses and improve how such analyses are reported to build confidence in models (page 18):

“Reporting guidelines for simulation modeling could set out common language for discussing sensitivity and uncertainty analyses, specify what methods details should be in the main text of an article versus in supplementary material (e.g., time horizon, time step used for discrete models), and set standards for reporting and discussing model assumptions.”

Third, we very much agree that studies should be more explicit in how they evaluate parameters used in their model, aligned with best practices for transparency and validation of models (broadly, building confidence in models, page 18). In our experience, simulation modeling studies of the type reviewed here do not specify causal estimand(s) because their main focus is on prediction, typically using the results of prior inferential studies (e.g., behavioral studies of warning labels, estimates of price elasticities). This means that assumptions about the causal strength of parameters are ‘baked into’ the model. We think that modelers are generally aware of the limitations of parameter estimates in their work and how the strength of those parameter estimates influences the overall strength of their model, but that thought process may not be included in published papers. We have added some additional language to this point on page 15:

“Including these details is important both to establish confidence in modeled results (e.g., if there are concerns about the causal strength or appropriateness of parameter estimates used)…”

2. Along with these methodological perspectives, it would then be useful to summarize how uncertainties were related to stakeholder challenges of implementation (i.e., the degree by which implementation of policy in different contexts might need to mitigate against potential uncertainties in impact). This is already well described (e.g., lack of details on how tax rates would be implemented, affecting robustness of the simulation models), but being non-specific with respect to each policy consideration, a summary of all studies on the extent by which issues of implementation in relation to model uncertainties are considered and addressed would be useful.

   Such an overarching summary would then reflect the extent by which simulation studies do or do not fully embrace complexities in policy implementation across different settings, and reveal to what degree simulation studies are mindful of uncertainties in relation to implementation by first recognizing such an issue and then dealing with it as part of the modelling process, with potential advice for stakeholders engaged in subsequent implementation.

   **R2.2**. We have refined our language to help make these conclusions clearer. We agree that it is crucial for policy simulation models to help decision makers understand how the realities of contexts, etc. will impact realized effects during implementation.

   To bolster our discussion of this important point, we have made the following changes:

   First, we have included a more direct overview of how *both* tax and non-tax policy impacts were simulated (pages 8 and 9, with an additional citation to a randomized behavioral science study that informed a warning label simulation (#82)):

   “Simulations of these policies used estimated impacts from published behavioral science research or assumptions about behavioral responses to these changes. For example, a warning label simulation model used observed effects on purchases of SSBs from a randomized trial in a mock convenience store [43,82]. Another model simulated the consumption effects of a portion size policy by assuming any modeled individual who drank a beverage larger than the portion size cap in the policy would reduce their consumption after policy implementation to drink exactly the portion size specified in the policy [35].”

   Second, we modified the discussion to focus more on policy implementation considerations (pages 14-15). In particular, we changed language in a summary paragraph to be applicable to both tax and non-tax policies:

   “Broadly, future modeling research should seek to be attentive to real-world policymaking and implementation questions. Modeling results will be more useful for policy implementation when researchers clearly define tax and non-tax policies and include implementation details in their models, including the scope of regulated SSBs and associated implementation scenarios. Models can be used to probe how different contexts impact policy implementation, or how industry responses to policy implementation could impact policies’ realized health effects [33]. With an eye towards informing policymaking and implementation, engaging stakeholders will be critical to ensure that models have the best chance to inform advocacy efforts and contribute to policymaking and implementation.”

   Third, we note how sensitivity analyses are important to “both to establish confidence in modeled results… and to help policymakers understand what to expect under different implementation scenarios” (page 15, with additional citation (#105)):

   “Including these details is important both to establish confidence in modeled results (e.g., if there are concerns about the causal strength or appropriateness of parameter estimates used) [104] and to help policymakers understand what to expect under different implementation scenarios [105].”
